# Immediate full weightbearing with additive cerclage improves early mobility after tibial shaft spiral fractures

**DOI:** 10.1038/s41598-025-24566-8

**Published:** 2025-10-23

**Authors:** Leonard Lisitano, Timon Röttinger, Stefan Eger, Carl Neuerburg, Edgar Mayr, Stefan Förch

**Affiliations:** 1 Department for Trauma, Orthopedics, Hand and Plastic Surgery, University Hospital Augsburg, Stenglinstr. 2, 86156 Augsburg, Germany; 2 Department for Orthopedics and Trauma Surgery, Musculoskeletal University Center Munich (MUM), University Hospital LMU Munich, Munich, Germany

**Keywords:** Tibial shaft spiral fractures, Additive cerclage, Immediate full weightbearing, Orthopedic surgery, Gait analysis, Return to work, Musculoskeletal system, Bone

## Abstract

In recent years, the trend in orthopedic and trauma surgery has shifted towards earlier mobilization with reduced weightbearing restrictions to enhance patient recovery. Tibial-shaft-spiral-fractures pose a challenge for early mobilization. This study examines the use of additive cerclages, which allow for immediate full-weightbearing, and compares it to the standard-of-care (SOC) treatment with partial-weightbearing. The primary aim was to evaluate mobility, return-to-daily-activities, and safety outcomes. This prospective multicenter study included 36 patients with tibial-shaft-spiral-fractures. Group1 (*n* = 20) received an additive cerclage and was permitted immediate full weightbearing-as-tolerated, while Group2 (*n* = 16) followed SOC with 20 kg partial weightbearing for 6 weeks. Gait analysis was performed at 1,3,6, and 12-weeks, as well as 6-months post-surgery, using the loadsol^®^ force-measuring device. Patient-reported outcomes, including pain levels, quality of life (EQ-5D), and return to daily activities, were assessed at these intervals. The results showed that immediate weightbearing as tolerated led to significantly faster mobilization. By week-3, 35% (7 of 20) of patients in Group1 were walking unassisted compared to 6.25% (1 of 16) in Group 2 (*p* = 0.04). By week 6, 75% (15 of 20) of patients in Group1 were walking without crutches, compared to 31.25% (5 of 16) in Group2 (*p* = 0.002). Gait speed and mobility were significantly improved in the cerclage group throughout the early postoperative period, with quality of life scores also higher during the early postoperative phase (gait speed week 3 2.57 ± 0.49 km/h vs. 2.16 ± 0.70 km/h, *p* = 0.032; EQ-5D week 1 3.59 ± 0.85 vs. 3.05 ± 0.56, *p* = 0.017). No significant differences in complications were observed between the groups. The use of additive cerclages for tibial-shaft-spiral-fractures is a safe and effective method that allows for immediate full-weightbearing, resulting in earlier mobilization and improved short-term quality of life.

Trail registration number: DRKS00035464 (www.drks.de), Date of registration: 17/12/2024.

**Supplementary Information:**

The online version contains supplementary material available at 10.1038/s41598-025-24566-8.

## Introduction

In recent years, there has been a noticeable shift within orthopedic and trauma surgery towards earlier mobilization and a reduction in weightbearing restrictions^1,2^. Numerous studies have explored treatment modalities that enhance mobilization following various injuries^3,4^. Despite this progress, standard of care (SOC) procedures still impose significant weightbearing restrictions for many injuries, particularly those affecting the lower limb. These restrictions predominantly impact vulnerable areas such as the tibial plateau, tibial shaft, and ankle. While the benefits of early mobilization and weightbearing are now commonly known, surgeons must carefully manage the risk of osteosynthesis failure, which could occur with immediate loading of the limb.

The standard of care (SOC) treatment for spiral-type fractures of the tibial shaft includes either intramedullary nailing or plate osteosynthesis^5^. According to the AO guidelines, these fractures are typically managed with 10–12 weeks of weightbearing restrictions, limiting the load to 15 kg^6^. However, adding a cerclage to the osteosynthesis can enhance biomechanical stability sufficiently to permit immediate weightbearing as tolerated^7^. Although the minimally invasive cerclage technique at the tibial shaft has been known for over 90 years, it remains relatively uncommon^8^. This reluctance largely stems from concerns about the potential entrapment of nerves or blood vessels and the fear that bone healing could be adversely affected. Nevertheless, there is no scientific evidence to substantiate these concerns^9^.

Furthermore, recent studies have indicated that early weightbearing may shorten time to union without increasing complication rates, but clinical data remain limited. A systematic review and meta-analysis reported that early weightbearing after intramedullary nailing of tibial shaft fractures was associated with a shorter mean union time and fewer total complications compared with delayed weightbearing^10^.

The aim of this study is to compare the effects of adding cerclages, which enable immediate weightbearing as tolerated, in tibial shaft spiral fractures. Outcomes will be assessed in terms of pain levels, gait patterns, return to work/sports, and the incidence of complications, in comparison to the standard of care (SOC) treatment.

## Methods

**Fig. 1:**
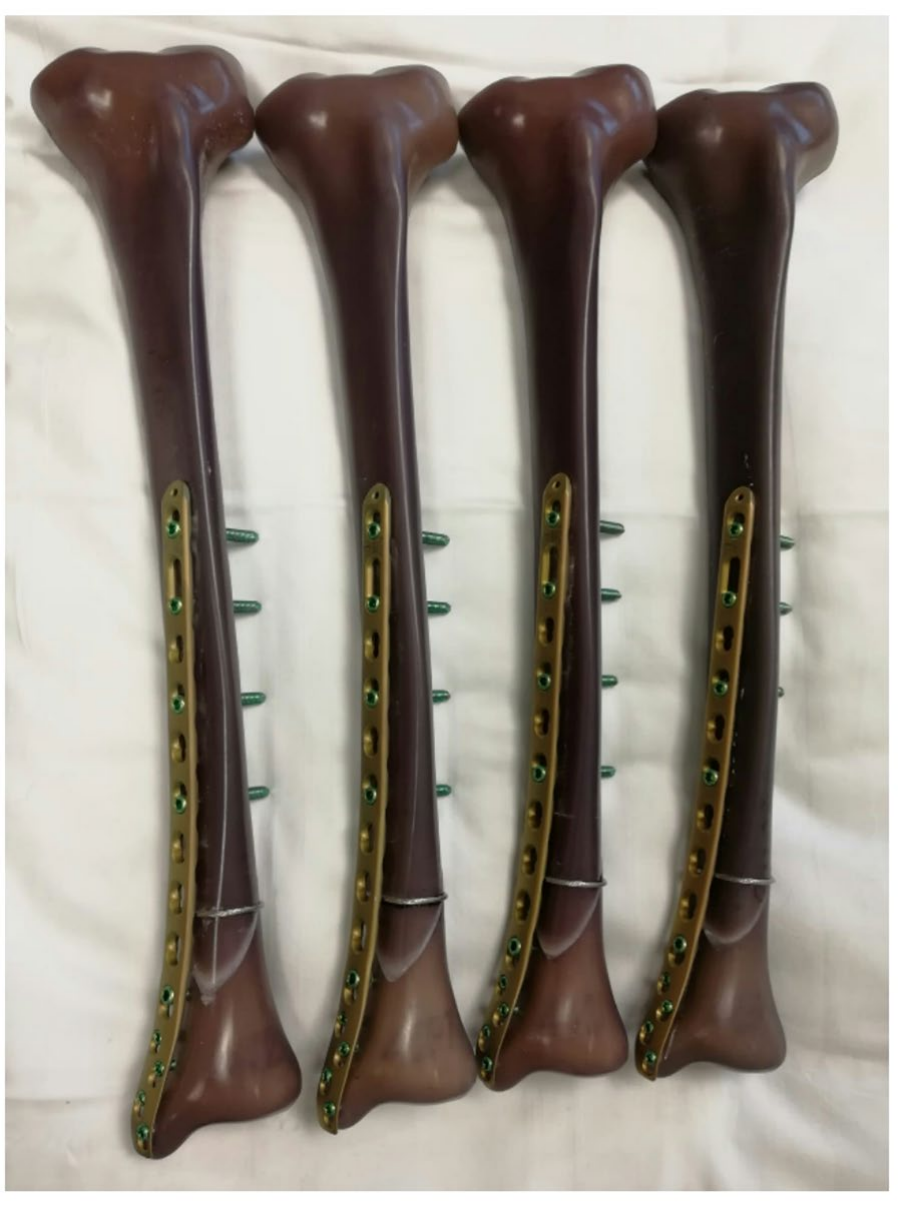
Shows osteosynthesis with a locking plate and an additional cerclage in four artificial tibiae. These models were used for biomechanical testing. In real patients, screws were measured and inserted at an appropriate length (bicortical, avoiding excessive protrusion).

In this prospective pseudo-randomized multicenter trial, a total of 36 patients who met the inclusion and exclusion criteria were enrolled. The study was conducted at two comparably sized university hospitals located close to each other. At one hospital, all eligible patients received treatment with additional cerclages and were allowed immediate full weightbearing as tolerated. In contrast, the other hospital did not use cerclages, and patients followed partial weightbearing restricted to 20 kg for 6 weeks. Other than that, all patients followed the same rehabilitation protocol. The experience level of the surgeons, as well as the annual number of surgeries performed, was very similar across both hospitals. The pseudo-randomization/grouping was determined by the hospital to which the patient was admitted. Figure 1 illustrates osteosynthesis with a locking plate and additional cerclage.

Participants eligible for this study were aged between 18 and 65 years and presented with tibial shaft spiral fractures classified as either AO:42A1 or AO:43A1. All included patients were treated with either plating (locking plate) or intramedullary nailing during the study period. Additionally, patients were required to complete follow-up at the admitting hospital. Written informed consent was obtained from all participants.

Patients were excluded from the study if they had illnesses that impair mobility, such as hemiparesis, or were unable to walk unassisted prior to the accident. Furthermore, individuals with cognitive impairments affecting their ability to comply with weightbearing restrictions, such as dementia, were also excluded.

Eligible patients were approached for consent in person by trained and certified research staff within two days following their final surgery; some patients may have received temporary external fixation prior to this. Baseline data, imaging results, and records of any complications were extracted from patient charts. Additionally, all participants underwent a gait analysis at 1, 3, 6, and 12 weeks, as well as 6 months post-surgery. Concurrently, patients completed questionnaires assessing pain, physical function, and quality of life at these same intervals. All collected data were stored pseudonymously. Following the conclusion of data collection six months post-surgery, the data was irreversibly anonymized.

Gait analysis was conducted using the loadsol^®^ force measuring device (Novel GmbH, Germany). Patients were instructed to walk a short course designed to mimic their typical home environment, using any assistive devices they normally would, such as crutches. The course incorporated several activities: standing up from a chair, walking, turning, sitting down on a chair, and climbing five stairs. During these activities, all ground reaction forces and gait speeds were recorded.

This study was conducted in accordance with the ethical standards laid down in the 1964 Declaration of Helsinki and its later amendments. Approval for the study was granted by the local ethics committee.

A power analysis was conducted to determine the required sample size. Assuming a significance level (α) of 0.05 and a power of 80%, a minimum of 15.5 patients per group was required to detect a mean decrease of three weeks in the time to walking without an assistive device (full weightbearing).

Statistical analysis was conducted using SPSS version 28 (IBM, Germany). An exploratory data analysis was performed to investigate the dataset. For nominal variables, the Chi-Square test and Fisher’s exact test were utilized to assess the associations. For metric variables, multilinear regression and multiple correlation analyses were employed to explore relationships and predict outcomes.

## Results

### Descriptive statistics

A total of 36 patients were included in the study, with Group 1 (*n* = 20) receiving an additional cerclage and immediate full weightbearing, while Group 2 (*n* = 16) followed the standard of care (SOC) with partial weightbearing. The mean age of participants in both groups was similar (Group 1: 48.65 ± 14.26 years, Group 2: 48.75 ± 15.46 years). Gender distribution was comparable, with 8 females and 12 males in Group 1, and 6 females and 10 males in Group 2. The average body weight was 648.25 ± 122.89 N in Group 1 and 640.24 ± 169.20 N in Group 2. A propensity score was calculated for each patient based on age, sex, fracture type, ASA classification, and comorbidities. The mean propensity scores were comparable between groups (Group 1: 0.44 ± 0.05; Group 2: 0.45 ± 0.08; *p* = 0.422). In Group 1, 16 patients underwent locking plate osteosynthesis, while 4 had intramedullary nailing. In Group 2, 4 patients received locking plate osteosynthesis, and 12 underwent intramedullary nailing.

### Time to walk unassisted

The duration until patients were able to walk without assistive devices (such as crutches) varied between the two groups. In Group 1 (cerclage with immediate full weightbearing), 2 out of 20 patients (10%) were walking unassisted after one week. By week 3, 7 out of 20 patients (35%) in Group 1 achieved unassisted walking. In contrast, none of the patients in Group 2 (standard of care with partial weightbearing) were able to walk without crutches at week 1, and 1 out of 16 patients (6.25%) reached this milestone by week 3 (Although partial weightbearing of 20 kg was prescribed for the first 6 weeks in this group). The differences in the proportion of patients walking unassisted between the groups were statistically significant at week 3 (*p* = 0.04).

By week 6, 15 out of 20 patients (75%) in Group 1 were able to walk without crutches, compared to 5 out of 16 patients (31.25%) in Group 2, with a statistically significant difference between the groups (*p* = 0.002). At week 12, 19 out of 20 patients (95%) in Group 1 were walking unassisted, compared to 11 out of 16 patients (68.75%) in Group 2 (*p* = 0.059). By 6 months postoperatively, all patients in both groups were able to walk without assistive devices.

### Gait analysis and peak force

**Fig. 2:**
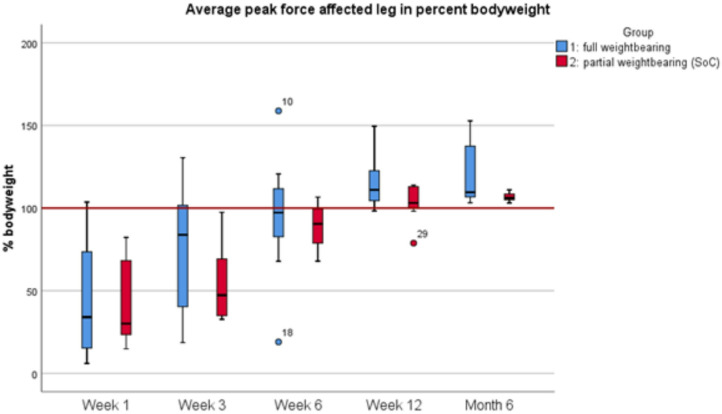
The boxplots in Fig. 2 illustrate the load on the operated leg as a percentage of body weight at various time points postoperatively. The y-axis represents the percentage of body weight applied, with the red line at 100% indicating full weightbearing.

The average peak force (APF) applied to the operated limb in Group 1 increased over time. At postoperative week 1, the APF was 348.73 ± 254.34 N, which increased to 825.85 ± 163.65 N by 6 months. In Group 2, the APF followed a similar pattern but remained lower in the first 12 weeks, with an initial value of 274.22 ± 165.59 N at week 1, increasing to 834.29 ± 232.07 N at 6 months. Differences were not statistically significant. The average peak force over time for both groups are presented in Fig. 2.

### Speed and mobility

At week 1, no significant differences in gait speed were observed between the groups (*p* = 0.459). However, by week 3, Group 1 exhibited a significantly faster gait speed (2.57 ± 0.49 km/h) compared to Group 2 (2.16 ± 0.70 km/h, *p* = 0.032). This trend continued, with Group 1 maintaining faster speeds at later time points, although the differences were not always statistically significant (Week 6: *p* = 0.08; Week 12: *p* = 0.073). At 6 months, Group 1 had a significantly higher speed (5.30 ± 1.02 km/h) than Group 2 (3.44 ± 0.86 km/h, *p* = 0.038).

### Complications and fracture consolidation

The rate of complications was low and comparable between the groups. In Group 1, one patient experienced a superficial wound infection unrelated to the cerclage (Infection 1/20, overall complication rate 5%). In Group 2, two patients developed wound infections, one of which required surgical revision (Infection 2/16, overall complication rate 12.5%). After 6 months, all patients in both groups showed adequate healing of the fracture, and no cases of malunion were observed.

### Compliance with weightbearing restrictions for group 2

In Group 2, where a 20 kg partial weightbearing restriction was prescribed for the first 6 weeks, compliance was limited. At week 1, 2 out of 16 patients (12.5%) maintained the prescribed load under 20 kg. An additional 4 patients (25.0%) carried loads between 20 kg and 30 kg, which was considered an acceptable overload. However, 5 patients (31.25%) exceeded this limit more than twice, applying over 40 kg on the affected limb. By week 3, none of the patients maintained the load under 20 kg. Five patients (31.25%) managed to keep their weightbearing between 20 kg and 30 kg, while 9 patients (56.25%) exceeded 40 kg.

### Influence of implant type

To assess whether implant type influenced the main outcomes, additional analyses were conducted. No significant differences were found between plate and nail fixation regarding time to full weightbearing, return to work or sports, or average peak force. Only gait speed at 3 and 6 weeks showed slightly higher values for plate fixation (*p* = 0.047 and *p* = 0.044). A two-way ANOVA including implant type (plate vs. nail) and its interaction with treatment group confirmed that implant type had no significant effect on time to full weightbearing (*p* = 0.478), and no significant interaction between implant type and group was observed (*p* = 0.666). The treatment group effect remained borderline significant (*p* = 0.050).

### Patient reported outcomes

No significant differences were found between the groups in terms of pain (VAS scores), either at rest or during weightbearing activities. However, subjective quality of life, as measured by the EQ-5D, was reported to be significantly better in Group 1 during the partial weightbearing period (week 1: *p* = 0.017; week 3: *p* = 0.045). By week 6, these differences had subsided, with no significant differences observed between the groups thereafter.

Additionally, the time to return to work and return to sports was shorter in the cerclage group. The mean time to return to work in Group 1 (cerclage) was 12.78 ± 7.06 weeks, compared to 17.25 ± 6.15 weeks in Group 2, with a near-significant difference (*p* = 0.057). Similarly, the return to sports occurred earlier in Group 1, with a mean time of 11.56 ± 6.31 weeks, versus 15.38 ± 7.26 weeks in Group 2, though this difference was not statistically significant (*p* = 0.123).

## Discussion

The primary finding of this study is that the use of additive cerclage in conjunction with intramedullary nailing or plating for tibial shaft spiral fractures enables immediate full weightbearing, resulting in significantly earlier mobilization without assistive devices compared to the standard of care (SOC). Immediate full weightbearing, permitted by the additional stability provided by the cerclage, also facilitated a faster increase in the load applied to the affected limb. Over the first three months postoperatively, the cerclage group consistently demonstrated higher peak forces on the operated leg compared to the SOC group.

Moreover, gait speed was significantly faster in the cerclage group throughout the early postoperative phase, with notable differences observed through week 12. These clinical benefits appear to be particularly pronounced in the initial weeks following surgery, a critical period for patient recovery and return to normal activities.

These findings align with previous research, such as a meta-analysis on ankle fractures which reported that early weightbearing led to improved outcomes, particularly between 6 and 9 weeks post-surgery^1,11,12^. Similar to our study, the meta-analysis found no significant differences between groups at 6 months, indicating that the primary advantage of early weightbearing lies in the early postoperative period. In that study, patients in the early weightbearing group also returned to work and daily activities significantly faster, a trend mirrored in our findings^1^.

In addition to the benefits of faster mobilization, the present study also found that quality of life, as measured by the EQ-5D, was significantly lower in the SOC group during the period of weightbearing restrictions. This reduction in life quality may be attributed to the restricted mobility imposed on the SOC group, as well as their slower recovery in terms of walking unassisted. The limited ability to bear weight and move independently is likely a key factor negatively impacting their perceived quality of life during this critical recovery period.

Importantly, the data also showed that there were no significant differences in pain levels or medication usage between the two groups, suggesting that pain did not have a substantial influence on the observed differences in quality of life. This further supports the hypothesis that it was the mobility limitations, rather than pain, that primarily contributed to the lower quality of life in the SOC group during the first six weeks post-surgery.

These findings highlight the significant role that early mobilization plays in improving both physical outcomes and overall well-being during the recovery process. As other studies on early weightbearing protocols have shown, faster recovery of mobility tends to correlate with improved quality of life, particularly during the early stages of rehabilitation^13–15^.

Another notable finding in the present study is the low compliance with weightbearing restrictions in Group 2. Despite being instructed to perform partial weightbearing (20 kg limit) for the first six weeks, many patients exceeded this limit. In fact, one patient was already walking without crutches after just three weeks. This non-compliance is consistent with existing literature, which reports that adherence to weightbearing restrictions is often poor in orthopedic recovery, particularly when patients are unsupervised^16^.

Studies have shown that many patients find it difficult to comply with strict weightbearing limitations, even when monitored by physical therapists​^16^. Factors such as age, cognitive ability, and a lack of proper feedback on weightbearing can all contribute to non-compliance^17^. Moreover, in everyday settings without close medical supervision, compliance is often even lower​.

Given this low compliance, the negative impact of weightbearing restrictions on life quality and daily activities, as observed in Group 2, may be underestimated. It is possible that if all patients adhered strictly to the 20 kg restriction, the effects on mobility, independence, and quality of life would have been even more pronounced.

In terms of complications, there were no significant differences between the two groups. The rate of wound infections, implant failure, or any other postoperative complications remained comparable, regardless of whether a cerclage was used. This aligns with the current literature, which does not suggest any increase in complication rates specifically associated with the use of cerclages in tibial shaft fractures​^8,9^.

In fact, studies examining the safety of cerclage techniques in orthopedic procedures have found it to be a reliable and effective method for enhancing stability without increasing the risk of adverse outcomes^7^. The concerns regarding potential neurovascular entrapment or impaired bone healing, often cited in historical discussions of cerclage, are not supported by contemporary research^9^.

Thus, the findings from this study reinforce the notion that the use of cerclage is a safe adjunct in fracture management, allowing for early full weightbearing without an elevated risk of complications.

One limitation of the present study is the relatively low number of patients included, which could potentially affect the generalizability of the findings. However, despite the small sample size, the study still yielded statistically significant results, particularly in terms of early mobilization, gait speed, and peak force measurements. These significant outcomes suggest that the sample size was sufficient to answer the main research questions regarding the efficacy and safety of additive cerclage in enabling early full weightbearing. Another limitation could be the different distribution of implant types between the groups. However, additional testing using a two-way ANOVA showed that implant type and its interaction with treatment group had no significant effect on the main outcomes, indicating that implant choice did not confound the study results. Nevertheless, the absence of a statistically significant difference does not necessarily imply equivalence between groups. The higher proportion of plate fixations in the early weightbearing group may have contributed to faster walking recovery, as plate osteosynthesis avoids intraarticular invasion around the knee joint compared to intramedullary nailing. Therefore, a potential influence of implant type on the observed outcomes cannot be entirely excluded.

In terms of strengths, the study had a rigorous design with several key elements enhancing its reliability. First, the follow-up period of 6 months provided enough time to assess both short-term recovery and medium-term functional outcomes. Second, the regular and standardized gait analysis conducted at 1, 3, 6, and 12 weeks, as well as 6 months, allowed for a detailed and consistent evaluation of patient mobility and weightbearing progression. Finally, the pseudo-randomization based on the admitting hospital helped minimize selection bias, as patients were allocated to treatment groups without deliberate manipulation.

To our knowledge, this is the first study to specifically examine and compare the use of additive cerclages at the tibial shaft, including detailed gait data and outcomes at the 6-month mark. This adds valuable insight into the use of cerclage in fracture management, particularly in relation to its impact on early mobility and long-term recovery.

## Conclusion

The use of additive cerclages in tibial shaft spiral fractures is a safe and effective method that enables immediate weightbearing as tolerated. This significantly enhances early mobility, accelerates the return to daily activities, and improves quality of life in the short-term postoperative period. The benefits of early mobilization are clear during the first weeks of recovery, while long-term outcomes remain comparable to the standard of care. Therefore, additional cerclages with immediate full weightbearing offer a valuable approach for improving early functional recovery without compromising safety.

## Supplementary Information

Below is the link to the electronic supplementary material.

Supplementary Material 1

## Data Availability

The datasets used and/or analyzed during the current study are available from the corresponding author on reasonable request.

## References

[1] Sharma, T. & Farrugia, P. Early versus late weight bearing & ankle mobilization in the postoperative management of ankle fractures: A systematic review and meta-analysis of randomized controlled trials. *Foot Ankle Surg. Off J. Eur. Soc. Foot Ankle Surg.* **28**, 827–835. 10.1016/j.fas.2022.03.003 (2022). 10.1016/j.fas.2022.03.003 35337752

[2] Dong, W., Lisitano, L. S. J., Marchand, L. S., Reider, L. M. & Haller, J. M. Weight-bearing guidelines for common geriatric upper and lower extremity fractures. *Curr. Osteoporos. Rep.* **21**, 698–709. 10.1007/s11914-023-00834-2 (2023). 37973761 10.1007/s11914-023-00834-2

[3] Bando, K. et al. Early weight bearing and mobilization decrease perioperative complications in patients after ankle fracture; the retrospective multicenter (TRON group) study. *J. Orthop. Sci. Off J. Jpn Orthop. Assoc. [Internet] J. Orthop. Sci.*10.1016/j.jos.2022.03.002 (2023). [cited 2024 Oct 10];28. 10.1016/j.jos.2022.03.002 35370043

[4] Tong, J. et al. Early versus delayed weight bearing and mobilization after ankle fracture fixation surgery: A systematic review and Meta-analysis of randomized controlled trials. *Orthopedics* **47**, 71–78. 10.3928/01477447-20230804-08 (2024). 37561102 10.3928/01477447-20230804-08

[5] White, R. & Camuso, M. Oct. Treatment of Simple fracture, spiral [Internet]. AO Surg. Ref. [cited 2024 Oct 10]. (2024). https://surgeryreference.aofoundation.org/orthopedic-trauma/adult-trauma/tibial-shaft/simple-fracture-spiral. Accessed 10.

[6] White, R. & Camuso, M. Oct. ORIF - Lag screws through protection plate for Simple fracture, spiral [Internet]. AO Surg. Ref. [cited 2024 Oct 10]. (2024). https://surgeryreference.aofoundation.org/orthopedic-trauma/adult-trauma/tibial-shaft/simple-fracture-spiral/orif-lag-screws-through-protection-plate. Accessed 10.

[7] Sandriesser, S. et al. Supplemental cerclage wiring in angle stable plate fixation of distal tibial spiral fractures enables immediate post-operative full weight-bearing: a Biomechanical analysis. *Eur. J. Trauma. Emerg. Surg. Off Publ Eur. Trauma. Soc.*10.1007/s00068-020-01503-0 (2020). 10.1007/s00068-020-01503-0 PMC8825397 32989509

[8] Iván, I. & Károly, K. [Experience with the treatment of longitudinal oblique and spiral fractures of the leg by goetze’s percutaneous cerclage]. *Magy Traumatol. Orthop. Helyreallito Seb*. **18**, 223–227 (1975). 240089

[9] Förch, S., Sandriesser, S., Fenwick, A. & Mayr, E. Beeinträchtigung der Blutversorgung durch cerclagen: mythos Oder Realität? Ein Überblick über die experimentelle studienlage. *Unfallchirurg* **124**, 231–240. 10.1007/s00113-020-00847-x (2021). 32813053 10.1007/s00113-020-00847-x PMC7921058

[10] Bhanushali, A. et al. Outcomes of early versus delayed weight-bearing with intramedullary nailing of tibial shaft fractures: a systematic review and meta-analysis. *Eur. J. Trauma. Emerg. Surg.* **48**, 3521–3527. 10.1007/s00068-022-01919-w (2022). 35238986 10.1007/s00068-022-01919-w PMC9532312

[11] Dehghan, N. et al. Early weightbearing and range of motion versus Non-Weightbearing and immobilization after open reduction and internal fixation of unstable ankle fractures: A randomized controlled trial. *J. Orthop. Trauma.* **30**, 345–352. 10.1097/BOT.0000000000000572 (2016). 27045369 10.1097/BOT.0000000000000572

[12] Flowers, D. W., McCallister, E., Christopherson, R. & Ware, E. The safety and effectiveness of Early, progressive weight bearing and implant choice after traumatic lower extremity fracture: A systematic Review. Bioengineering. *Multidisciplinary Digit. Publishing Inst.* **9**, 750. 10.3390/bioengineering9120750 (2022). 10.3390/bioengineering9120750 PMC9774827 36550956

[13] de Sa, R., Shah, N., Rudge, B. & Ieong, E. Safety of early weightbearing after ankle fracture fixation. *Eur. J. Orthop. Surg. Traumatol.* **34**, 1003–1007. 10.1007/s00590-023-03758-w (2024). 37843568 10.1007/s00590-023-03758-w

[14] Canton, G., Sborgia, A., Dussi, M., Rasio, N. & Murena, L. Early weight bearing in tibial plateau fractures treated with ORIF: a systematic review of literature. *J. Orthop. Surg.* **17**, 261. 10.1186/s13018-022-03156-8 (2022). 10.1186/s13018-022-03156-8 PMC9097122 35549974

[15] Pfeufer, D. et al. Weight-bearing restrictions reduce postoperative mobility in elderly hip fracture patients. *Arch. Orthop. Trauma. Surg.* **139**, 1253–1259. 10.1007/s00402-019-03193-9 (2019). 31053870 10.1007/s00402-019-03193-9

[16] Eickhoff, A. M. et al. Analysis of partial weight bearing after surgical treatment in patients with injuries of the lower extremity. *Arch. Orthop. Trauma. Surg.* **142**, 77–81. 10.1007/s00402-020-03588-z (2022). 32880704 10.1007/s00402-020-03588-z PMC8732824

[17] Seo, H. et al. Factors affecting compliance with Weight-Bearing restriction and the amount of Weight-Bearing in the elderly with femur or pelvic fractures. *Ann. Rehabil Med. Korean Acad. Rehabilitation Med.* **44**, 109–116. 10.5535/arm.2020.44.2.109 (2020). 10.5535/arm.2020.44.2.109 PMC7214136 32392649

