# Supplementary material for: Immediate full weightbearing with additive cerclage improves early mobility after tibial shaft spiral fractures
Source: Sci Rep. 2025 Oct 23;15:37166. doi: 10.1038/s41598-025-24566-8 (PMC12549978; doi:10.1038/s41598-025-24566-8)
Supplement: Supplementary file 1 — Supplementary Material 1 [file 41598_2025_24566_MOESM1_ESM.pdf]

# Testing Protocol

## Calibration and Device Preparation

All loadsol® insoles were calibrated weekly according to the manufacturer's specifications. Per the manufacturer's guidance, individual calibration for each patient was not required. Before each testing session, the appropriate insole size was selected for each participant. After insertion into the patient's footwear, participants were instructed to walk a few meters to allow the sensors to adapt. Subsequently, the insoles were zeroed under full unloading to ensure accurate baseline measurement.

## Testing Environment

All measurements were conducted under standardized environmental conditions with optimal lighting. Testing took place on a predefined indoor course and was supervised by trained personnel experienced in gait analysis using loadsol® sensors.

## Test Procedure

Participants began the test in a seated position on a standardized, stable chair with fixed armrests. The sequence was as follows:

1. **Sit-to-Stand and Walking Task:**
   The participant stood up independently from the chair and walked 10 meters in a straight line, turned around freely, and returned to the chair. Upon completion, the participant sat down again.
2. **Stair-Climbing Task:**
   After the walking task, the participant ascended and descended a standardized five-step staircase constructed according to DIN norms. A single handrail, compliant with standard dimensions, was available on one side.

At the end of the stair task, the measurement was stopped.

## Standardization and Instructions

All tests were carried out by trained medical staff following a standardized procedure. The test protocol was demonstrated and explained to each participant before their first measurement. No verbal instructions or corrections were given during the test itself to avoid influencing natural gait behavior.

A detailed *"Handbuch des klinischen Prüfers / Clinical Investigator's Manual"* (in German) describing all testing steps and safety considerations is available upon reasonable request.
